# Modification of soybean growth and abiotic stress tolerance by expression of truncated ERECTA protein from *Arabidopsis thaliana*

**DOI:** 10.1371/journal.pone.0233383

**Published:** 2020-05-19

**Authors:** Sudha Shanmugam, Shan Zhao, Soumen Nandy, Vibha Srivastava, Mariya Khodakovskaya

**Affiliations:** 1 Department of Biology, University of Arkansas at Little Rock, Little Rock, Arkansas, United States of America; 2 Department of Crop, Soil, and Environmental Sciences, University of Arkansas, Fayetteville, Arkansas, United States of America; RIKEN Center for Sustainable Resource Science, JAPAN

## Abstract

*ERECTA* gene family encodes leucine-rich repeat receptor-like kinases that control major aspects of plant development such as elongation of aboveground organs, leaf initiation, development of flowers, and epidermis differentiation. To clarify the importance of ERECTA signaling for the development of soybean *(Glycine max)*, we expressed the dominant-negative *ERECTA* gene from *Arabidopsis thaliana* that is truncated in the kinase domain (*AtΔKinase*). Expression of *AtΔKinase* in soybean resulted in the short stature, reduced number of leaves, reduced leaf surface area and enhanced branching in the transgenic plants. The transgenic *AtΔKinase* soybean plants exhibited increased tolerance to water deficit stress due to the reduction of total leaf area and reduced transpiration compared to the wild-type plants. Production of seeds in *AtΔKinase* lines was higher compared to wild type at regular conditions of cultivation and after exposure to drought stress. Transgenic seedlings expressing *AtΔKinase* were also able to withstand salt stress better than the wild-type. Established results demonstrated the significance of native soybean genes (*GmER* and *GmERL*) in development and stress response of soybean, and suggested that the truncated *ERECTA* gene of *Arabidopsis thaliana* can be used to manipulate the growth and stress response of different crop species.

## Introduction

One of the major questions of developmental biology is how the body and organ size of multicellular organisms is controlled by intrinsic factors [1] The *ERECTA* gene family of leucine-rich repeat receptor-like kinases (LRR-RLK) is a pleiotropic regulator of various developmental processes [2]. In *Arabidopsis thaliana*, the synergistic action of three *ERECTA*-gene family, *ERECTA(ER)*,*ERECTA-LIKE 1(ERL1)* and *ERECTA-LIKE 2(ERL2)*, controls aboveground organ growth and flower development. These genes regulate shoot apical meristem size, help to establish phyllotaxy, and promote cotyledon [3,4]. *Arabidopsis thaliana ERECTA* gene family inhibits the differentiation of the protodermal cells into meristemoid mother cells, thereby preventing the formation of guard mother cells [3]. Further, through expression in the epidermis, *ERECTA* gene family controls stomatal formation. These genes decrease the stomatal density in leaves, reducing the overall stomatal conductance and the water loss [5].

ERECTA signaling has been extensively studied. It has been demonstrated that the *ERECTA* family receptors are localized in the plasma membrane, where they sense small extracellular peptide [6]. The *ER* family receptors after perceiving the secreted peptide ligands, epidermal patterning factor 1(EPF1) and epidermal patterning factor 2 (EPF2) modify stomatal patterning [7–9]. Recently, two more peptides (EPF4 and EPF6) were also identified as ligands in the signaling process [10,11]. Shpak et al., (2005) suggested that TMM (TOO MANY MOUTHS) LRR-receptor-like protein forms a heterodimer with the *ER* family RLK and prevents signaling, thereby negatively regulating stomatal differentiation. A MAP kinase cascade consisting of YODA (YDA), MKK4/MKK5 and MPK3/MPK6 operate downstream of ER receptors [12]. It was suggested that MAPKc, cascade involved in regulating inflorescence architecture is based on both gain- and loss-of-function data [13–15].

Up to date, the functions of *ERECTA* gene family were mostly characterized in *Arabidopsis*, which is considered as a model plant for all genetic studies. ERECTA plays an important role in regulating the size of the aboveground organs of *Arabidopsis* [14]. Loss of function of ERECTA leads to plants with short stature and tightly clustered inflorescences which is due to the reduced internode and pedicel lengths [16]. Also mutant *ERECTA (er)* plants of *Arabidopsis* also display shorter hypocotyls, smaller cotyledons, and leaves with short petioles and wider flowers with short and blunt siliques, with a reduced number of cortex cells in the stems and pedicels but more expanded cells than the wild-type [6,13,15,17,18]. The truncated ERECTA protein that lacks the cytoplasmic kinase domain *(ΔKinase)* confers dominant-negative effects in *Arabidopsis* including compact inflorescence and short, blunt siliques [13]. Analysis of the *ERECTA* gene family phylogenetic tree suggested that this group of genes are quite conserved between *Arabidopsis* and other plant species [25]. Thus, *Arabidopsis er* phenotypes serve as evidence that modifications of ERECTA signaling in valuable crop species could lead to desirable phenotypical traits that can be achieved through overexpression or suppression of *ERECTA* genes.

However, studies of *ERECTA* gene family other than *Arabidopsis* plants are still limited. Involvement of an *ER* homolog in *Sorghum bicolor*, *SbRLK1* in the regulation of specific processes in the mesophyll cells was reported [19]. In *Zea mays*, *ERECTA* genes play a role in controlling plant growth, organ size and yield of plants [20]. Lately, ERECTA signaling in *Solanum lycopersicum* was manipulated by expressing *AtΔKinase* from *Arabidopsis* using two different promoters [25]. Thus, the expression of *AtΔKinase* under the control of *35S* promoter dramatically reduced vegetative growth and production of seeds in the transgenic tomato lines [25]. Expression of *AtΔKinase* under the control of its own promoter, on the other hand, resulted in relatively moderate inhibition of tomato plant height but significant decrease in the number of leaves and total leaf area [25].

Several research studies [21, 22, 23, 25] reported links between ERECTA signaling and plant response to abiotic stress. For example, two homologs of *ERECTA* in the *Triticum aestivum* genome, *TaER1* and *TaER2*, were recently connected with the improvement of transpiration efficiency and yield in bread wheat [21]. The role of *ERECTA* gene family in regulating thermotolerance in *Oryza sativa* and tomato has recently been characterized [22]. Thermotolerance was reduced in a loss-of-function *ER* homolog rice mutant and tomato with reduced expression of a tomato *ER* allele [22]. Lately, it has been shown that an *ER* homolog in wild common bean might be associated with drought [23]. Authors reasoned that the reduction of total leaf area (evaporating surface area) in the transgenic tomato plants, caused by the expression of *AtΔKinase*, conferred drought tolerance [25]. Most importantly, these transgenic tomato lines did not suffer yield loss as determined by fruit size and number per plant. Described traits will be very desirable for many crops including soybean (*Glycine max*). Du et al., [24] found that several soybean *ER* homologs can be upregulated by water stress. Based on the sequence similarity between *Arabidopsis ER* genes and the predicted soybean *ER* and *ERL* genes [25], it is logical to assume that *Arabidopsis* and soybean ER signaling pathways are quite conserved. Therefore, *Arabidopsis ER* gene can be used to manipulate the development and stress response in soybean as was previously documented for tomato plants [25].

Here, we determined the role of ERECTA signaling in the development and stress response of soybean plants by disrupting ERECTA signaling using the transgenic approach. Soybean lines expressing the truncated ERECTA protein from *Arabidopsis* (*AtΔKinase*) were established and analyzed. *AtΔKinase* expressing transgenic soybean lines exhibited short stature, reduced leaf area, and a significant increase in tolerance to increased salinity and water deficit stress. We demonstrated that the establishment of more compact and stress-tolerant crop plants with no yield penalty can be achieved successfully by suppressing ERECTA signaling.

## Materials and methods

### Vector construction and transgenic soybean lines development

The 8.018 kb *Eco*R1 –*Bam*H1 fragment from pESH454 consisting of *Arabidopsis ERECTA* gene truncated in the region encoding the kinase domain (*AtΔKinase*) [13] was cloned into soybean transformation vector pTF101.1 [26] obtained from Iowa State University using the standard cloning techniques. The truncated *ER* gene in pNS37 contains native Arabidopsis *ER* (At2g26330) promoter (1.8 kb), gene fragment (4.2 kb), and the native transcription terminator (1.9 kb). The 6 kb EcoR1 –BamH1 fragment from pESH454 was cloned between EcoR1 and BamH1 sites of pTF101.1 followed by the introduction of 2 kb BamH1 fragment to build pNS37. The *ER* gene fragment consists of exons and introns for LRR repeats and transmembrane (TM) region. There is a stop codon immediately after TM sequence followed by BamH1 site and *ER* terminator. The pTF101.1 vector contains 2 x 35S promoter-driven Bar gene as the selection marker (S1 Fig). The resulting pNS37 vector was submitted to Iowa State University for developing transgenic soybean lines using the Williams 82 genotype, where *Agrobacterium*-mediated transformation protocol [27,28] was pursued to develop putative transgenic lines.

### Expression analysis of transgene and native soybean *ERECTA* genes using real-time RT-PCR

Total RNA was isolated from the apex and young leaves of 21-day-old soybean plants, flowers from 35-d-old mature plants and siliques of 45-d-old plants using an RNeasy Plant Mini kit (Qiagen, Germany). The cDNA was generated from 1μg of total RNA using a SuperScript III First-Strand Synthesis System (Invitrogen, USA) with a dT20 oligonucleotide as primer according to the manufacturer’s protocol. cDNA samples were diluted and used for real-time quantitative PCR analysis with SYBR Green PCR master mix (Thermo Fisher Scientific, UK) in an iCycler iQ Multicolor Real-Time PCR detection system (Bio-Rad, USA). *At*Δ*Kinase* gene was amplified using primers: 5’-ATGGTATGACATTTGACTCCAAAC -3’ and 5’-GTGGATCTATTCCTCCGATTCTC -3’. House-keeping gene (18S) was amplified using primers: 5’-AGGCCGCGGAAGTTTGAGGC-3’ and 5’-ATCAGTGTAGCGCGCGTGGG -3’. The primers used to amplify the native *GmERL1* gene (NP_001237639) were: 5’-GCTCGGAATAGGCTCAGTGG -3’(Forward) and 5’-ACGATATGTCCAGGTACTGCAA-3’(Reverse). To amplify the native *GmERL2* gene (NP_001235330), 5’-CTAGTGGAACTGGGCAAGG-3’(Forward) and 5’-TGGGTGGCCATAATAATACTAAGCA-3’(Reverse) primers were used. The native *GmERL3* gene (XP_003534036) was amplified using the primers: 5’-TGTTGGCTTTTGTGGGCAAG-3’(Forward) and 5’-TCGCTGAGTGGTGAAGCAAA-3’(Reverse). House-keeping gene (18S) was amplified using primers: 5’-AGGCCGCGGAAGTTTGAGGC-3’(Forward) and 5’-ATCAGTGTAGCGCGCGTGGG -3’(Reverse). Three independent biological replicates were used in the analysis. For each biological replica, three technical replicas were run. The real-time PCR data analyzed by the ‘comparative count’ method to obtain relative mRNA expression of each tissue as described in the iCycle manual (Bio-Rad).

### Plant growth conditions and phenotypical analysis of transgenic soybean plants

Seedlings (10-day-old) of wild-type and the selected transgenic soybean lines (3–1, 2–1, 2–4 and 1–2) were transferred into small pots containing sterile Sun Shine Redi-earth Professional growing mix. All plants were grown in a growth chamber under conditions of 12 h light (25 °C) and 12 h dark (20 °C), 45% humidity, and 500 μmol m–2 s–1 light intensity and were watered once a day. Three-week-old seedlings were transferred to the greenhouse in a bigger pot. Seven mature plants from each experimental group (WT, 3–1, 2–1, 2–4 and 1–2) were phenotypically analyzed, and the following data were recorded: length of the leaves, the total number of leaves and number of branches in 66-d-old plants, number of mature siliques and number of seeds in 80-day-old plants. One-way ANOVA (Analysis of Variance) with post-hoc Tukey HSD (Honestly significant difference) using SAS software (SAS Studio 3.8) [29] was used to assess the significance of differences in the data between groups of plants. In a separate experiment, plant height was recorded every 5 days starting from 30-day-old plants, up to the state of full maturity (70 days). Total leaf surface area was measured in 66-day-old plants by removing all the leaves from a plant and then measuring the area using the portable leaf area meter android [Biovis Leaf Av (Android version), Expert Visions Labs Pvt. Ltd, India].

### Water loss assay and drought stress experiment

To examine the ability of transgenic plants to lose water, three flag leaves were excised from each 4-week-old wild-type and transgenic plants grown in the greenhouse, and the fresh weight was immediately determined. All the leaves were placed on a laboratory bench at room temperature for 300 min. Every 30 min, leaf weight was recorded. Water loss was calculated as the percentage of the initial fresh weight at each time point. For each genotype (WT, 3–1, 2–1, 2–4 and 1–2), five plants were tested. The relative water content of the detached leaves was measured by the leaf disc method [30]. Six leaves were cut from each 4-week plant grown in the greenhouse. Leaf discs were made from each leaf with the help of a cork borer and fresh weight was immediately measured. The discs were dipped in distilled water and kept in the refrigerator (4 °C) for 24 hours to reach full turgor. The turgid weight and later the dry weight after drying the discs at 70°C for 24 hours was measured. The RWC in % was calculated by the formula: RWC (%) = [(fresh weight-dry weight)/(turgid weight-dry weight)] x100. To test drought tolerance at the adult stage, wild type and a transgenic line were grown under normal watering conditions for 4 weeks and then subjected to drought stress by withdrawing irrigation for the next 6 weeks. After 14 days of drought stress, drought tolerance phenotypes were examined, and pictures of transgenic lines and wild-type were captured. A total of 10 plants for each of WT and transgenic lines were evaluated in water deficit experiment. During the drought stress experiment, the volumetric water content of the soil was measured at 0, 5, 7 and 9 days using the ProCheck decagon device (Decagon Devices, Inc., USA). The photosynthetic activity and the stomatal conductance was also measured using a portable photosynthesis system (Li-Cor LI-6400XT) (LI-COR Biosciences, USA). Three measurements were made for each plant, and 7 plants were used for both the wild type and the transgenic plants.

### Salt stress experiments

To estimate the working concentration of NaCl for salt stress experiments, the wild type seeds were grown on both Murashige and Skoog medium (MS) without NaCl supplement (control) and MS media supplemented with different concentrations of NaCl (50mM, 100mM, 150mM, 200mM, 300mM, and 400mM). The shoot and root lengths, as well as fresh and dry biomass of shoots and roots of 10-day-old germinated seedlings, were measured and the data were analyzed statistically using One-way ANOVA (Analysis of Variance) with post-hoc Tukey HSD (Honestly significant difference) using SAS software. In the salt experiment involving transgenic lines, the seeds of wild type and transgenic lines were germinated in the presence of NaCl. Seeds were surface-sterilized using chlorine gas in a desiccator and planted on both pure MS medium (control) and medium supplemented with 100 mM NaCl. Seeds were germinated under continuous light and the germination rate was recorded daily for 10 days. To test salt tolerance at the seedling stage, 10-day old seedlings were photographed to visualize the phenotypes. The root and shoot lengths, as well as whole fresh and dry biomass, were also measured. All tests were repeated thrice, and One-way ANOVA (Analysis of Variance) with post-hoc Tukey HSD (Honestly significant difference) using SAS software was used for statistical analysis.

## Results

### Development of transgenic soybean plants expressing *AtΔKinase* transgene

**Fig 1:**
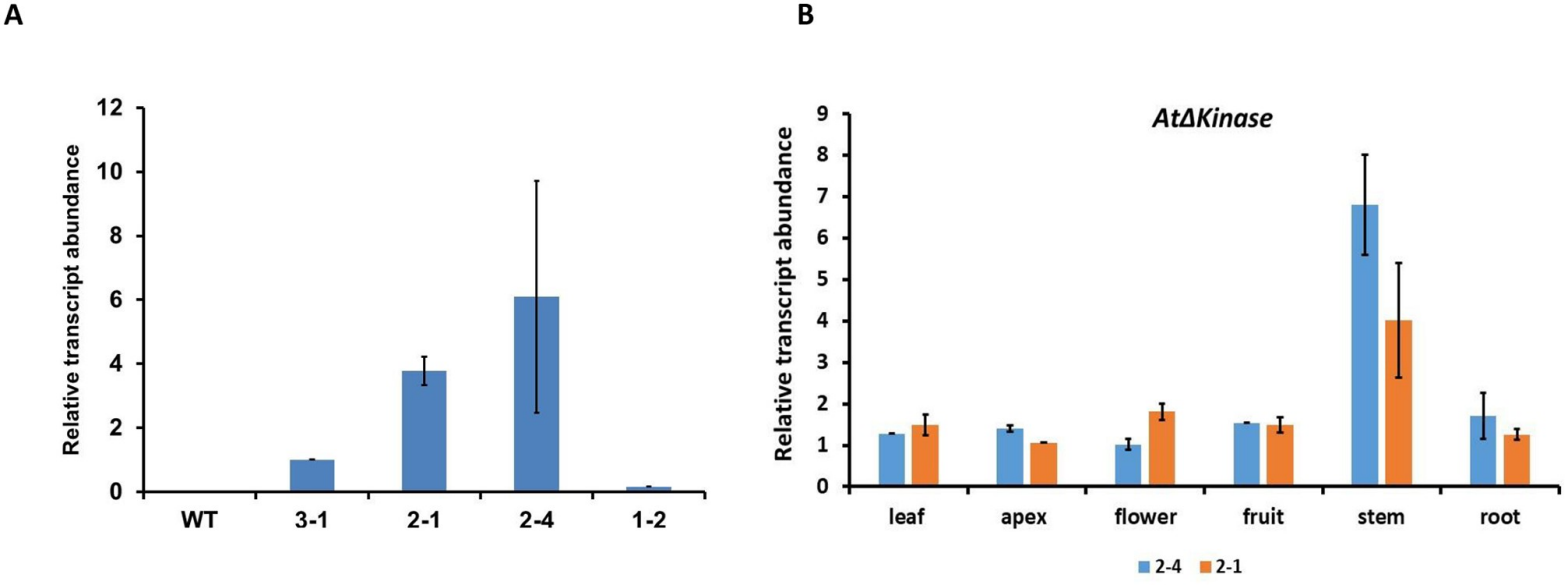
Analysis of *AtΔKinase* gene expression in the four generated transgenic lines (A) and different organs of selected *AtERpro*:*AtΔKinase* lines (B). The analysis was performed using quantitative RT-PCR. 18S was used as an internal control. For A- Samples collected from 14-day-old plants. For B- Leaves, apex, roots, stems were collected from 21-day-old plants, flowers were collected from 35-day-old plants, siliques were collected from 45-day-old plants. Results are shown as means ±SE of three biological replicates.

To disrupt ERECTA signaling in soybean, we took the dominant-negative approach and expressed a truncated version of the *Arabidopsis ERECTA* gene (*AtΔKinase*) under the control of native *ER* promoter *(AtERECTApro*::*AtΔKinase)* in soybean plants. The truncated ERECTA binds with its ligands and forms heterodimers; however, due to the deletion in the kinase domain, it is unable to phosphorylate and trigger signaling [13]. Therefore, the expression of *AtΔKinase* is expected to suppress ER signaling. A similar approach was successfully used in the tomato model plant [25], where expression of *AtΔKinase* resulted in drastic modification of tomato phenotype, including dwarfing of tomato plants and reduction in total leaf area. To determine the effect of *AtΔKinase* expression in soybean (cv. Williams 82), T_1_ seeds of 4 putative independent lines established as a result of transformation with *AtERpro*:*A*t*ΔKinase* plasmid were obtained from Plant Transformation Facility, Iowa State University. PCR analysis on germinated T_1_ seedlings of each line showed the presence of the *A*t*ΔKinase* gene. Low transformation efficiency can be explained by the strong effect of *AtΔKinase* transgene resulting in developmental defects. The expression of the transgene (*AtΔKinase*) in four established lines was confirmed by qPCR analysis (Fig 1A). The highest level of *AtΔKinase* was found in two transgenic lines (2–1 and 2–4). Both lines were selected for monitoring of *AtΔKinase* expression in different organs of transgenic lines (Fig 1B). It was confirmed that the *AtΔKinase* gene was expressed in leaves, apex, stem, root, flowers, and fruits of both selected lines. The highest expression of the transgene was observed in the stems of transgenic plants. Same transgenic lines (2–1 and 2–4) were used for the analysis of the expression of three native soybean *ERECTA* genes (*GmERL1*, *GmERL2*, *GmERL3)* in different organs (leaves, apex, stem, root, flowers, and fruits) of the wild type and *AtΔKinase* expressing lines (S2 Fig). We documented that the expression of all three native soybean *ERECTA* genes was enhanced in all analyzed tissues of the transgenic lines compared to wild type.

### Effects of suppression of ERECTA signaling on the phenotype of transgenic plants

**Fig 2:**
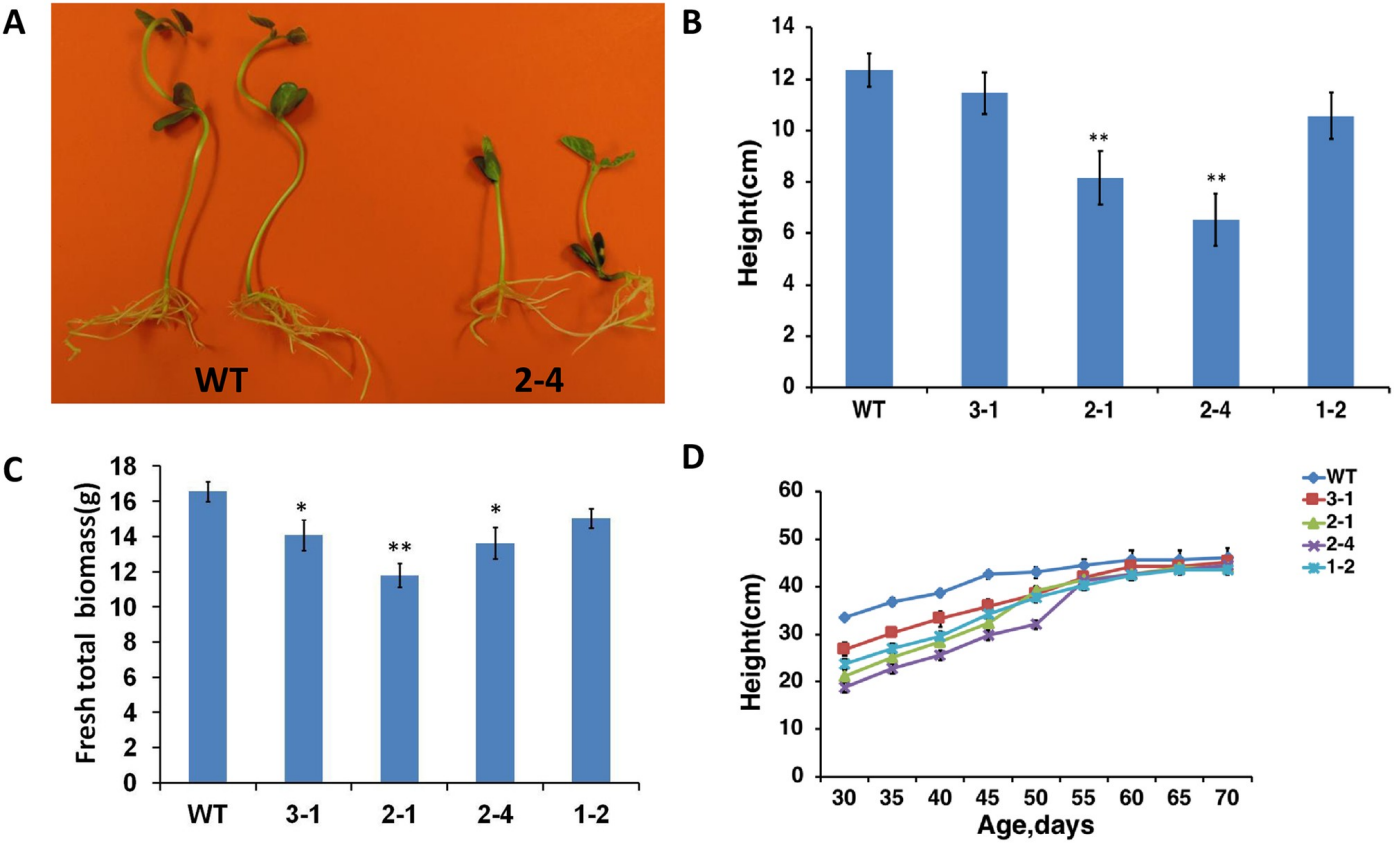
*AtERpro*:*AtΔKinase* transgenic soybean plants grow slowly compared with wild-type plants but can reach the same height at maturity. (A) Phenotypical comparison of 10-day-old seedlings of wild-type and *AtERpro*:*AtΔKinase* plants. (B) Height (n = 10) in 40-day-old wild-type soybean plants and *AtERpro*:*AtΔKinase* expressing lines. (C) Accumulation of fresh biomass (n = 6) of wild-type and *AtERpro*:*AtΔKinase* soybean transgenic lines after 90 days of cultivation in a growth chamber (D) Gradual changes in the height (n = 4) of wild-type and four transgenic soybean lines during 70 days of cultivation in a growth chamber. Results are shown as means ±SE (*P<0.05;**P <0.01). T1 generation of transgenic lines was analyzed in the presented phenotypical tests.

**Fig 3:**
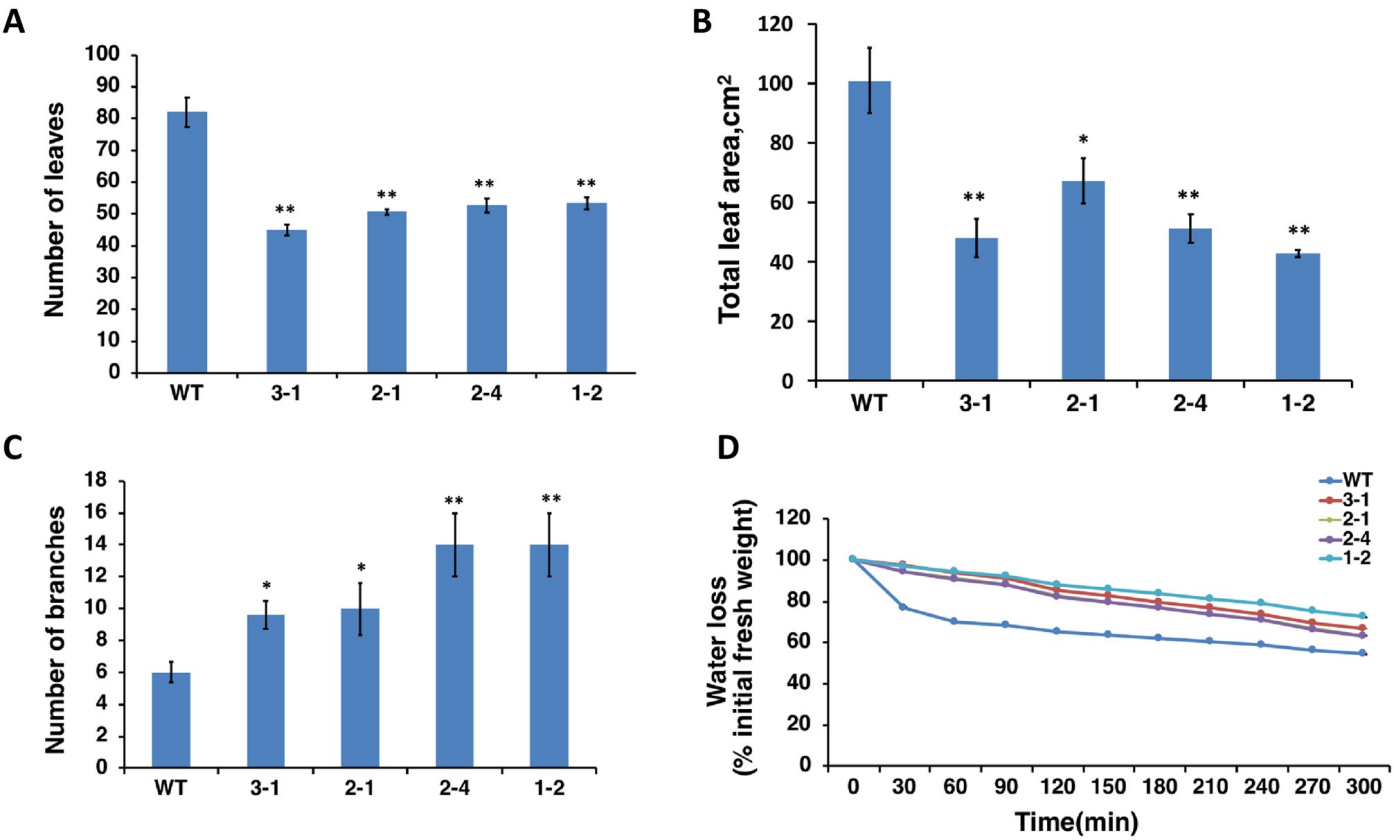
*AtERpro*:*AtΔKinase* transgenic plants generate less number of leaves (A) with smaller leaf area (B) but produce more branches (C) compared with wild-type soybean plants. Total leaf area per plant and a total number of leaves were calculated for 66-day-old wild-type and four *AtERpro*:*AtΔKinase* transgenic lines (n = 7; **P <0.01; *P <0.05). The number of branches was estimated for 62-day-old wild type and transgenic lines cultivated in greenhouse conditions. Water loss (D) in fully expanded and detached leaves was calculated for 58-day-old wild type and four transgenic lines. T3 generation of transgenic lines was used in stress experiments. Results are shown as means ±SE.

At the stage of young seedlings (10-day-old plants) and young plants (40-day-old), *AtERpro*:*AtΔKinase* expressing soybean lines grew slowly and appeared short compared to the wild-type (Fig 2A and 2B). As expected, the reduction in the size of transgenic plants resulted in a decrease in total biomass compared with the wild-type (Fig 2C). However, *AtERpro*:*AtΔKinase* lines reached the size of the wild-type plants at the maturity stage (Fig 2D). Thus, 70-day-old fully mature transgenic plants of four independent lines (3–1; 2–1; 2–4; 1–2) had the same height as the wild-type soybean plants (Fig 2D). At the same time, mature transgenic plants developed less number of leaves (Fig 3A) with decreased total leaf area per plant (Fig 3B) compared with the wild-type. On the contrary, the transgenic plants were found to have more branches (Fig 3C) than the wild-type, giving them short, compact and bushy appearance. It is important to note that *AtERpro*:*ΔKinase* soybean lines produced more seeds per plant than wild type. Thus, 80-day-old transgenic lines 3–1, 2–1, 2–4, 1–2 produced 12.5%, 39.2%, 23%, and 35.5% more seeds respectively compared to wild type plants of the same age (S3 Fig). Thus, we can conclude that the expression of a truncated version of *Arabidopsis ERECTA* (*AtΔKinase*) interfered with native soybean ERECTA signaling and inhibited the vegetative growth and development of transgenic soybean plants. These results were consistent with the results that were established for tomato expressing *AtΔKinase* [25]. It is interesting that the leaves detached from the *AtERpro*:*ΔKinase* transgenic lines were able to retain water and maintain initial weight than the leaves detached from the wild-type plants (Fig 3D). Previously, we reported that the disruption of ERECTA signaling in tomato plants through the expression of *Arabidopsis ΔKinase* led to significant improvement of drought tolerance in the transgenic tomato plants [25]. We also hypothesized that tolerance to water deficit was improved due to the considerable reduction of evaporating surface area (total leaf area) that may contribute to the reduction of transpiration in transgenic tomato plants expressing *AtΔKinase*. Since we have observed a decrease of leaf number and leaf area in *AtERpro*:*ΔKinase* transgenic soybean lines, we next addressed the response of *AtERpro*:*ΔKinase* transgenic lines on water deficit stress.

### Expression of *AtERpro*: *AtΔKinase* alters the response to water deficit stress in transgenic soybean plants

**Fig 4:**
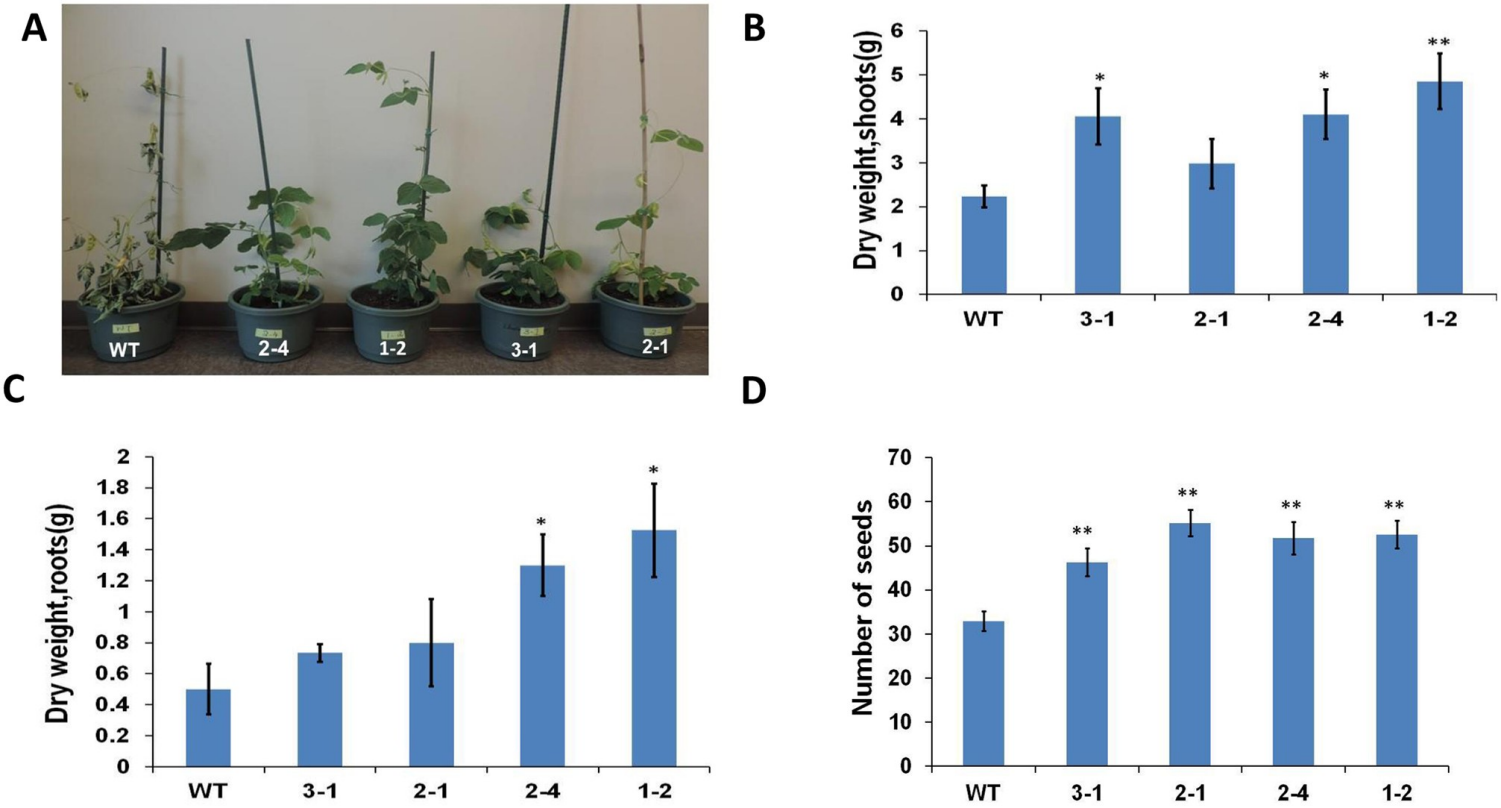
*AtERpro*:*AtΔKinase* transgenic plants are more tolerant to water deficit stress. 52-day-old *AtERpro*:*AtΔKinase* plants exhibited fewer visible stress symptoms after two weeks of incubation without watering (A). Dry biomass of shoots (B), roots (C) and the number of seeds (D) produced by 82-day-old wild type and transgenic lines after cultivation in water deficit conditions. n = 5; **P <0.01; *P <0.05. T_3_ generation of transgenic lines was used in stress experiments. Results are shown as means ±SE.

**Fig 5:**
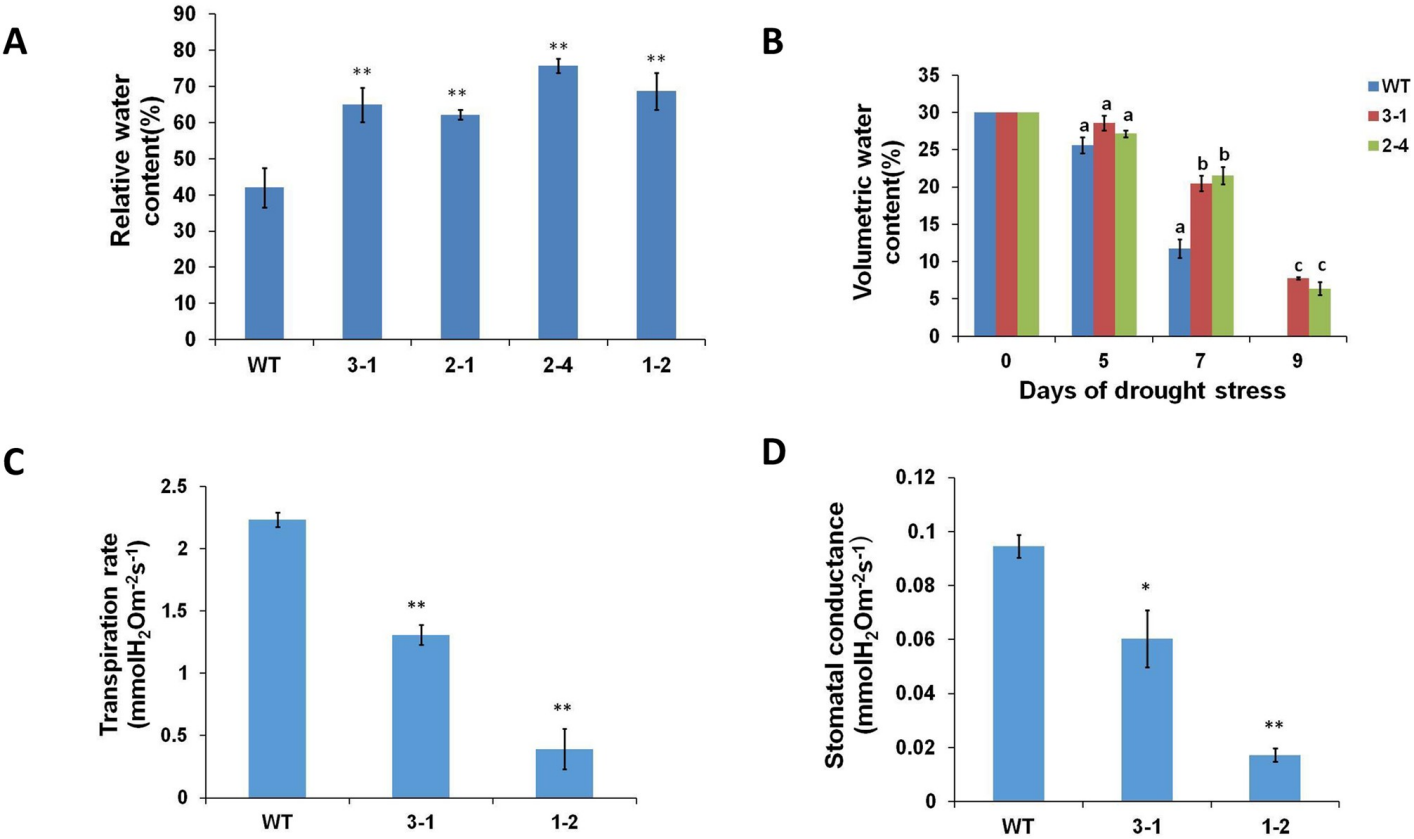
*AtERpro*:*AtΔKinase* transgenic plants transpire less water under water deficit conditions compared to the wild type. (A) Relative water content (leaves) in 58-day-old wild type and transgenic lines grown in regular greenhouse conditions. (B) Soil moisture expressed as volumetric water content (n = 7) in pots used for cultivation of wild-type and transgenic lines during 0, 5, 7 and 9 days of drought stress. Letter ‘a’ denote insignificant difference compared to that of wild type. Letters b and c denote significant differences compared to that of wild type. Letter b denotes p < 0.05, Letter c denotes p < 0.01 (C) Transpiration rate of wild-type and transgenic plants in day 7 of water deficit stress. (D) Stomatal conductance (n = 3) in leaves of wild type and transgenic *AtERpro*:*AtΔKinase* lines in day 7 of water deficit stress (n = 7). Results are shown as means ±SE.

We exposed *AtERpro*:*ΔKinase* transgenic soybean lines to water deficit stress by terminating water supply for 6 weeks. Soil moisture in pots was measured as volumetric water content (%) before the experiment and during the stress experiment. Initial volumetric water content (before water withholding) content was equal between pots used for the cultivation of wild-type and transgenic lines. After seven days of water termination, the wild-type plants exhibited typical symptoms of stress such as wilting and rolling of leaves, while the *AtERpro*:*AtΔKinase* transgenic lines appeared unstressed. The first symptoms of drought stress in the transgenic plants were observed after 2 weeks of drought stress, and such symptoms were less obvious compared to that in the wild-type (Fig 4A). At the end of stress experiment, the number of seeds per plant, as well as dry weight of the shoot and root biomass and number of produced seeds, was recorded (Fig 4B, 4C and 4D). We found that transgenic soybean lines produced more dry biomass of roots and shoots under water deficit conditions compared to the wild-type plants (Fig 4B and 4C). This can be explained by the ability of transgenic plants to retain water and continue growth for a longer time during the water withholding period than the wild-type control plants. Seed yield is the most valuable trait of soybean [31]. Previously, we have noticed that the expression of *AtΔKinase* in tomato plants did not cause a reduction of photosynthetic ability and suppression of the reproductive system (production of fruits/seeds per plant) [25]. Similarly, transgenic soybean plants expressing the same gene (*AtΔKinase*) and exposed to prolonged water deficit stress produced more seeds per plant compared with the wild-type (Fig 4D). To clarify the links between transpiration and the observed resistance to water deficit, we measured the stomatal conductance as well as the transpiration rate during the water deficit stress experiment (Fig 5). We did not detect any statistically significant differences in the number of stomata per leaf between wild-type and the transgenic lines (S4 Fig) However, both the stomatal conductance and transpiration rate were significantly decreased in the two selected transgenic lines (lines 3–1 and 1–2) compared to that of wild type plants (Fig 5C and 5D). As a result of decreased transpiration, the relative water content in the leaves was higher in *AtΔKinase* lines than in the wild-type (Fig 5A). To evaluate remaining soil moisture during the water deficit experiment, we measured volumetric water content in pots used for plant cultivation. As showed in Fig 5B, pots used for cultivation of transgenic plants maintained more moisture than pots with wild-type plants during the first 9 days of cultivation without watering. Based on these experimental data, we hypothesized that the increased drought tolerance of established transgenic lines is related to lower transpiration surface resulting from the smaller total leaf area of the transgenic *AtERpro*:*AtΔKinase* plants. To understand if ERECTA signaling is also linked with plant stress signaling pathways at the molecular level, we have selected two soybean genes *(GmRD22-Like* and *GmPIP1-2)* that can be used as markers for plant response to osmotic stress. *GmRD22-Like* is an apoplast‐localized BURP‐domain protein-encoding gene [42] and *GmPIP1-2* is the soybean water channel protein-encoding gene [44]. Expression of both selected genes was monitored by q-PCR in leaves of soybean wild type and all four *AtERpro*:*AtΔKinase* lines exposed to drought stress for seven days (S5 Fig). We have noticed a significant increase of expression of both genes in three transgenic lines (2–1; 2–4; 1–2) exposed to water deficit stress (S5 Fig).

### Expression of *AtERpro*:*AtΔKinase* results in enhancement of tolerance of transgenic soybean plants to salt stress

**Fig 6:**
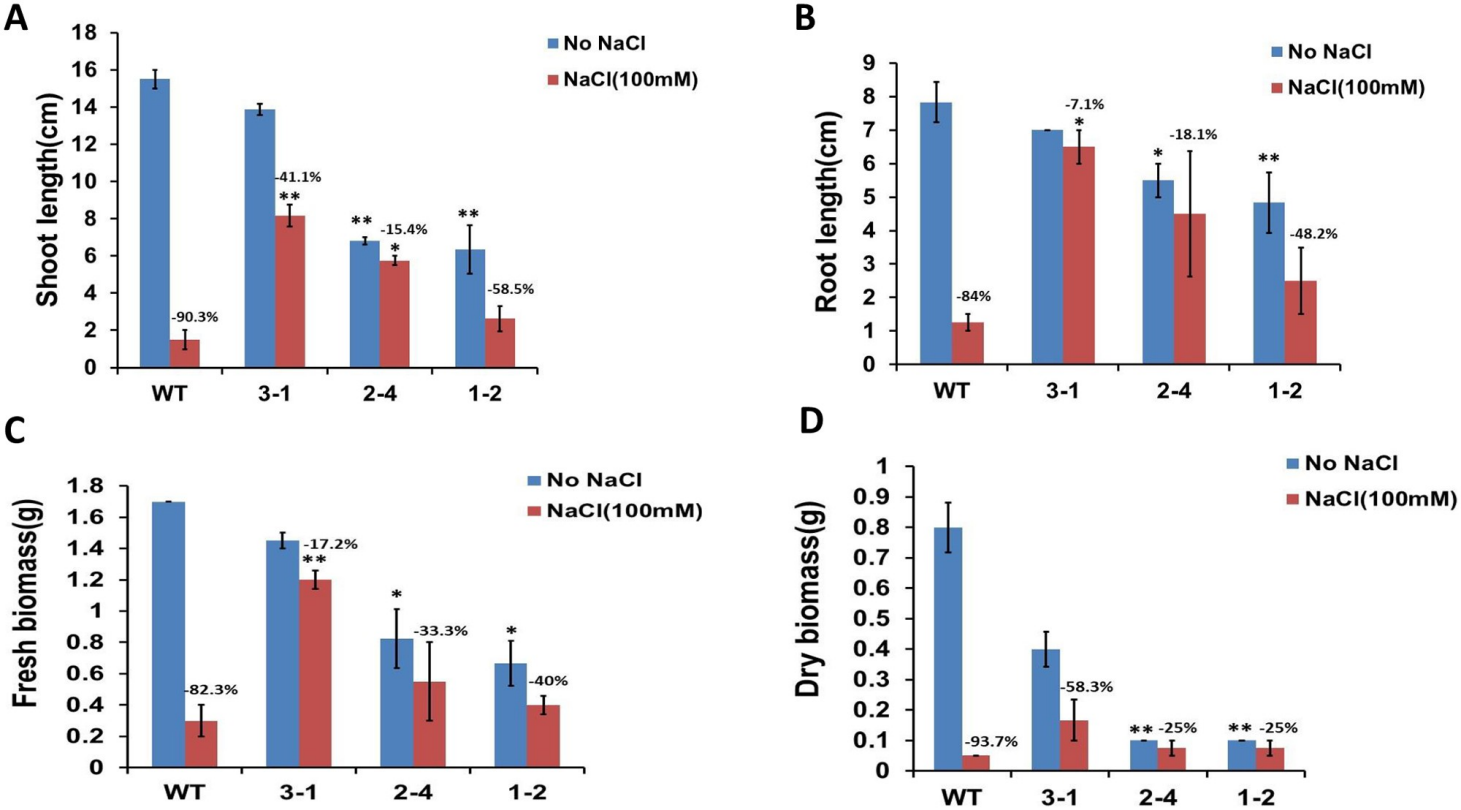
*AtERpro*:*AtΔKinase* transgenic soybean seedlings produce longer shoots, roots and total biomass than wild type plants under salt stress (NaCl, 100 mM). (A) Comparison of the shoot length in 10-day-old seedlings of wild type and transgenic lines grown under salt stress. (B) Comparison of the root length in 10-day-old wild type and transgenic seedlings grown under salt stress. (C) Comparison of the weight of total fresh biomass in the 10-day-old wild type and transgenic lines grown under salt stress. (D). Comparison of the weight of total dry biomass in the 10-day-old wild type and transgenic lines grown under salt stress. The seedlings (10-day-old) were grown both on regular MS medium (control) and in MS medium supplemented with NaCl (100mM) (n = 10; **P <0.01; *P <0.05). Results are shown as means ±SE.

To estimate the appropriate concentration of NaCl for salt stress experiments involving transgenic lines, NaCl in a wide range of concentrations was applied to the seeds of wild-type soybean (S6 Fig). 10-day-old wild-type seedlings were subjected for measurement of the length of shoots and estimation of the total biomass of NaCl-treated seedlings. Based on such measurements we found that NaCl in a concentration of 100 mM was toxic for soybean plants but the damage caused to the seedlings was less as seen at higher NaCl doses (S6 Fig). As the next step, seeds of wild-type and two *AtΔKinase* expressing lines (3–1,1–2) were exposed to regular MS medium and MS medium supplemented with 100mM NaCl. Seed germination rate, as well as accumulation of biomass of transgenic and control lines during 10 days of cultivation on regular MS medium and MS medium supplemented with salt, were recorded. As shown in Fig 6 and S7 Fig, *AtERpro*:*AtΔKinase* lines exhibited higher tolerance to salt stress compared to the wild-type. Thus, two selected transgenic lines (3–1 and 1–2) had a higher germination rate on NaCl-supplemented medium (S7 Fig). When the seeds were germinated and cultivated under salt stress (100 mM NaCl) for 10 days, *AtERpro*:*AtΔKinase* lines developed longer shoots and roots, and accumulated more total fresh and dry biomass than the wild-type seedlings exposed to the same salt stress (Fig 6A, 6B, 6C and 6D; S7 Fig). However, if seedlings were grown in regular MS medium (without NaCl supplement), transgenic plants were smaller and produced less biomass than wild-type seedlings (Fig 6A, 6B, 6C and 6D).

## Discussion

ERECTA is a leucine-rich repeat RLKs, which in turn are the plasma membrane-localized receptors that sense the extracellular signals in plants [32]. Phylogenetic analysis of the *ERECTA* gene family suggested that such a group of genes originated at an early stage of evolution. According to the constructed phylogenetic tree of the *ERECTA* family, three soybean *ERECTA* genes (*GmERL1*, *GmERL2*, *GmERL3*) are evolutionarily very close to *Arabidopsis* (*AtERL2*, *AtERL1*) and tomato (*SlERL*) *ERECTA* genes [25]. Thus, it is logical to expect the existence of functional similarity between *Arabidopsis*, tomato, and soybean *ERECTA* genes. Here, we took a dominant-negative approach to terminate soybean ERECTA signaling through the expression of *Arabidopsis thaliana ΔKinase* gene controlled with the *Arabidopsis* (*AtERpro*) promoter in soybean plants. The observed modification to architecture, productivity and stress response in *AtERpro*:*AtΔKinase* expressing soybean lines was very similar to changes that were earlier observed in *AtERpro*:*AtΔKinase* expressing tomato lines [25]. Transgenic soybean plants exhibited a significant reduction of height at a young stage and restored size at the stage of maturity. The total leaf number was decreased and branching was enhanced in the mature transgenic plants.

Similar to the *AtERpro*:*AtΔKinase* tomato plants [25], soybean *AtERpro*:*AtΔKinase* plants expressed *AtΔKinase* gene in all analyzed tissues including young leaves and apex. Interestingly, the expression of three soybean native *ERECTA* genes (*GmERL1*, *GmERL2*, *GmERL3)* was significantly enhanced in *AtERpro*:*AtΔKinase* soybean lines compared to the wild type (S2 Fig). This trend was noticed in all analyzed organs including leaf, apex, fruit, stem, and root. Similarly, native tomato *ERECTA* genes (*SlER and SlERL*) were overexpressed in *AtERpro*:*AtΔKinase* tomato plants compared to tomato wild type [25]. This observation can indicate that termination of ERECTA signaling by expression of *AtERpro*:*AtΔKinase* can lead to compensation effect through induction of native *ERECTA* genes.

It is well known that ERECTA signaling is associated with abiotic and biotic stress response [2]. The petioles in *Arabidopsis er* mutants are shorter compared to that of the wild-type, and in response to certain environmental changes like flooding, low light, and shade, *ERECTA* can promote hyponastic growth and petiole elongation due to its effect on petiole morphology [2, 33]. Overexpression of the *ERECTA* gene from *Populus nig*ra in *Arabidopsis* led to increased photosynthetic rate, decreased transpiration and increased water use efficiency [34]. ERECTA was also reported to protect against heat stress in *Arabidopsis* during an adaxial-abaxial polarity formation in the leaves [35], and contribute to disease resistance by reducing pathogen invasion and spread [2]. Such an increase of biotic stress tolerance is most likely linked to the impact of ERECTA on plant morphology, especially on the structure of epidermis and vasculature [2]. Recently, it was discovered that ERECTA interacts with BAK1 (BRASSINOSTEROID INSENSITIVE 1-associated kinase 1) receptor-like kinases, and regulates immune responses in *Arabidopsis* [36]. Overexpression of *ERECTA*, improved thermotolerance in rice and tomato [22]. On the contrary, *er* mutations led to decreased thermotolerance in both tested species [22].

The exact mechanism of links between abiotic stress signaling and ERECTA signaling is not clarified yet. Such a mechanism most likely is associated with drastic morphological changes observed in transgenic lines overexpressing *ERECTA* genes or in the *er* mutants. Indeed, it was reported that *er* mutation can alter leaf morphology including stomatal density, epidermal cell expansion, mesophyll cell proliferation, circadian leaf movements and petiole growth [5, 37, 38]. Other changes in leaf morphology caused by the termination of ERECTA signaling can also contribute to the modified response to environmental stress. Here, we have linked observed reduction of total leaf area in *AtΔKinase* transgenic soybean lines with a reduction of total transpiration and as a result, enhanced tolerance to water deficit stress (Figs 2, 3 and 4). Grain production is the major soybean trait. *AtΔKinase* expressing soybean lines produced more seeds per plant after long-term exposure to water deficit stress compared to the wild–type. Thus, genetic modification of ERECTA signaling can be successfully used for the improvement of grain-producing crops without a reduction in the yield. Observed improvement of salt stress tolerance of *AtERpro*:*AtΔKinase* is another beneficial trait of soybean plant with terminated ERECTA signaling (Fig 6). Salinity is one of the greatest environmental challenges and one of the best solutions is to create salt-tolerant cultivars [39]. It is predicted that about 50% of the available land will be affected by salinity by 2050 [40]. Taking in account our data demonstrating improvement of drought and salt tolerance of soybean *AtERpro*:*AtΔKinase* transgenic lines and early documented thermotolerance of transgenic tomato and rice lines overexpressing *Arabidopsis ER* gene [22], we can conclude that genetic manipulations with ERECTA signaling may significantly contribute to the creation of crop cultivars with enhanced tolerance to different types of osmotic stress. Here, we have provided experimental evidence that the observed enhanced tolerance of *AtERpro*:*AtΔKinase* soybean lines to water deficit stress (Fig 3) can be mainly associated with phenotypical modifications of transgenic leaves including the reduced leaf surface area. However, ERECTA signaling may also be associated with other stress signaling pathways. Although our main focus was not on studies of such molecular links, we have monitored the expression of two soybean stress-inducible genes (*GmRD-22 Like* and *GmPIP1-2*) in wild type and *AtERpro*:*AtΔKinase* transgenic lines grown under water deficit stress for seven days (S5 Fig). It is known that both selected genes are playing a critical role in the response of plants to osmotic stress and can be used for the improvement of abiotic stress tolerance using a genetic approach [41, 42, 43, 44]. Thus, studies have shown that the triggered ABA production during dehydration stress increases the expression of *GmRD22* gene (apoplast‐localized BURP‐domain protein), which protects the plant from major abiotic stresses [41]. Another study has revealed that the expression of *GmRD22* in *Arabidopsis thaliana* and rice during stress could strengthen the cell wall integrity by increasing the lignin contents [42]. There is evidence of the involvement of PIP1 genes (water channel genes) in plant stress tolerance by maintaining the uptake and movement of water in the plant body [43]. It has been shown that the overexpression of *GmPIP1-2* gene (soybean water channel protein) in soybean has increased the plant’s abiotic stress tolerance, promote plant growth and finally increase the soybean yields [44]. It is interesting that in our studies both genes (*GmRD22-Like* and *GmPIP1-2*) were overexpressed in the transgenic lines after seven days of water deficit stress (S5 Fig). Thus, it is logical to suggest that the termination of ERECTA signaling achieved in our *AtERpro*:*AtΔKinase* lines can drastically affect stress signaling pathways and various transcriptional factors. Such influence can contribute to the observed effects of *AtΔKinase* gene on the response of *AtΔKinase* transgenic soybean plants on osmotic stress. To clarify the molecular mechanisms of effects of *ERECTA* genes on plant stress response more mechanistic studies should be done in the future.

## Supporting information

S1 Fig Map of vector construction used for soybean transformation. The pNS37 construct in pTF101.1 backbone is shown. The 6 kb EcoR1 –BamH1 fragment from pESH454 was cloned between EcoR1 and BamH1 sites of pTF101.1 followed by introduction of 2 kb BamH1 fragment to build pNS37. The ER gene fragment consists of exons and introns for LRR repeats and transmembrane (TM) region. There is a stop codon immediately after TM sequence followed by BamH1 site and ER terminator. The pTF101.1 vector contains 2 x 35S promoter driven Bar gene as the selection marker.
(TIF)

S2 Fig A quantitative RT-PCR analysis of endogenous *GmERL1* (A), *GmERL2* (B) and *GmERL3* (C) gene expression in different plant organs of wild type plants and of transgenic lines. 18S was used as an internal control. Samples of leaves, apices, stem and root were collected from 21-day-old plants, flowers were collected from 35-day-old plants and siliques derived from 45-day-old plants. Results are shown as means ±SE of three biological replicates.
(TIF)

S3 Fig A production of seeds by 80-day-old wild type and transgenic lines grown under regular greenhouse conditions. n = 10; **P <0.01; *P <0.05. Results are shown as means ±SE.
(TIF)

S4 Fig Stomatal density in the leaves of seedlings of wild type and two transgenic *AtERpro*: *AtΔKinase* lines. Six leaves were sampled and analyzed for each line. Values are mean ± SE.
(TIF)

S5 Fig A quantitative RT-PCR expressional analysis of osmotic stress marker genes (A) *GmRD22-Like* (B) *GmPIP1-2* in the leaves of wild type and *AtERpro*:*AtΔKinase* transgenic lines grown under drought stress. Leaves were collected from 28-day-old wild type and of transgenic plants grown in conditions of water deficit for 7 days. 18S was used as an internal control. Results are shown as means ±SE of three biological replicates.
(TIF)

S6 Fig Phenotypical comparison of 10-day-old soybean wild-type seedlings unexposed to salt stress and seedlings exposed to different concentrations of NaCl (50mM, 100mM, 150mM, 200mM, 300mM and 400mM) (A, B, C) n = 10; **P <0.01; *P <0.05. Results are shown as means ±SE. (D) Photograph of 10-day-old wild type seedlings grown without NaCl supplement (control) and grown in medium supplemented with a wide range of NaCl concentrations.
(TIF)

S7 Fig (A) The phenotype of wild type and transgenic *AtERpro*: *AtΔKinase* seedlings (line 3–1) exposed to salt stress conditions (100mM NaCl). (B) *AtERpro*:*AtΔKinase* transgenic plants exhibited higher germination rate on medium supplemented with 100mM of NaCl compared to wild-type.
(TIF)

## References

[1] Gilbert SF. Developmental biology, the stem cell of biological disciplines. PLOS Biol. 2017;15: e2003691 10.1371/journal.pbio.2003691 29284160 PMC5761959

[2] Shpak ED. Diverse Roles of ERECTA Family Genes in Plant Development. J Integr Plant Biol. 2013;55: 1238–1250. 10.1111/jipb.12108 24016315

[3] Shpak ED, McAbee JM, Pillitteri LJ, Torii KU. Stomatal Patterning and Differentiation by Synergistic Interactions of Receptor Kinases. Science. 2005;309: 290–293. 10.1126/science.1109710 16002616

[4] Chen M-K, Shpak ED. ERECTA family genes regulate development of cotyledons during embryogenesis. FEBS Lett. 2014;588: 3912–3917. 10.1016/j.febslet.2014.09.002 25240196

[5] Masle J, Gilmore SR, Farquhar GD. The *ERECTA* gene regulates plant transpiration efficiency in *Arabidopsis*. Nature. 2005;436: 866–870. 10.1038/nature03835 16007076

[6] Torii KU, Mitsukawa N, Oosumi T, Matsuura Y, Yokoyama R, Whittier RF, et al The Arabidopsis ERECTA gene encodes a putative receptor protein kinase with extracellular leucine-rich repeats. Plant Cell. 1996;8: 735–746. 10.1105/tpc.8.4.735 8624444 PMC161133

[7] Hunt L, Gray JE. The Signaling Peptide EPF2 Controls Asymmetric Cell Divisions during Stomatal Development. Curr Biol. 2009;19: 864–869. 10.1016/j.cub.2009.03.069 19398336

[8] Hara K, Yokoo T, Kajita R, Onishi T, Yahata S, Peterson KM, et al Epidermal Cell Density is Autoregulated via a Secretory Peptide, EPIDERMAL PATTERNING FACTOR 2 in Arabidopsis Leaves. Plant Cell Physiol. 2009;50: 1019–1031. 10.1093/pcp/pcp068 19435754

[9] Lee JS, Kuroha T, Hnilova M, Khatayevich D, Kanaoka MM, McAbee JM, et al Direct interaction of ligand-receptor pairs specifying stomatal patterning. Genes Dev. 2012;26: 126–136. 10.1101/gad.179895.111 22241782 PMC3273837

[10] Abrash EB, Davies KA, Bergmann DC. Generation of Signaling Specificity in *Arabidopsis* by Spatially Restricted Buffering of Ligand–Receptor Interactions. Plant Cell. 2011;23: 2864–2879. 10.1105/tpc.111.086637 21862708 PMC3180797

[11] Uchida N, Shimada M, Tasaka M. Modulation of the balance between stem cell proliferation and consumption by ERECTA-family genes. Plant Signal Behav. 2012;7: 1506–1508. 10.4161/psb.22080 22990445 PMC3548882

[12] Meng X, Wang H, He Y, Liu Y, Walker JC, Torii K, et al A MAPK Cascade Downstream of ERECTA Receptor-Like Protein Kinase Regulates Arabidopsis Inflorescence Architecture by Promoting Localized Cell Proliferation. Plant Cell. 2012;24 10.1105/tpc.112.104695 23263767 PMC3556968

[13] Shpak ED, Lakeman MB, Torii KU. Dominant-Negative Receptor Uncovers Redundancy in the Arabidopsis ERECTA Leucine-Rich Repeat Receptor–Like Kinase Signaling Pathway That Regulates Organ Shape. Plant Cell. 2003;15: 1095–1110. 10.1105/tpc.010413 12724536 PMC153719

[14] Shpak ED, Berthiaume CT, Hill EJ, Torii KU. Synergistic interaction of three ERECTA-family receptor-like kinases controls Arabidopsis organ growth and flower development by promoting cell proliferation. Development. 2004;131: 1491–1501. 10.1242/dev.01028 14985254

[15] Woodward C, Bemis SM, Hill EJ, Sawa S, Koshiba T, Torii KU. Interaction of Auxin and ERECTA in Elaborating Arabidopsis Inflorescence Architecture Revealed by the Activation Tagging of a New Member of the YUCCA Family Putative Flavin Monooxygenases. Plant Physiol. 2005;139: 192–203. 10.1104/pp.105.063495 16126863 PMC1203369

[16] van Zanten M, Snoek LB, Proveniers MCG, Peeters AJM. The many functions of ERECTA. Trends Plant Sci. 2009;14: 214–218. 10.1016/j.tplants.2009.01.010 19303350

[17] Rédei GP. Supervital Mutants of Arabidopsis. Genetics. 1962;47: 443–460. 17248096 10.1093/genetics/47.4.443 PMC1210343

[18] Bowman J. Arabidopsis: An Atlas of Morphology and Development. Springer Science & Business Media; 2012.

[19] Annen F, Stockhaus J. SbRLK1, a receptor-like protein kinase of Sorghum bicolor (L.) Moench that is expressed in mesophyll cells. Planta. 1999;208: 420–425. 10.1007/s004250050577 10384732

[20] Guo M, Rupe M, Simmons C, Sivasankar S. The maize erecta genes for improving plant growth, transpiration efficiency and drought tolerance in crop plants. WO2008039709A2, 2008. https://patents.google.com/patent/WO2008039709A2/en

[21] Zheng J, Yang Z, Madgwick PJ, Carmo-Silva E, Parry MAJ, Hu Y-G. TaER Expression Is Associated with Transpiration Efficiency Traits and Yield in Bread Wheat. Ma W, editor. PLOS ONE. 2015;10: e0128415 10.1371/journal.pone.0128415 26047019 PMC4457575

[22] Shen H, Zhong X, Zhao F, Wang Y, Yan B, Li Q, et al Overexpression of receptor-like kinase ERECTA improves thermotolerance in rice and tomato. Nat Biotechnol. 2015;33: 996–1003. 10.1038/nbt.3321 26280413

[23] Darkwa K, Ambachew D, Mohammed H, Asfaw A, Blair MW. Evaluation of common bean (Phaseolus vulgaris L.) genotypes for drought stress adaptation in Ethiopia. Crop J. 2016;4: 367–376. 10.1016/j.cj.2016.06.007

[24] Du JunBo, Sun Xin, Sun MengYuan Jiang HengKe, Yan Li, Wan ChuanYin, et al Identification and expression analysis of ERECTA homologous genes in Glycine max. Int J Agric Biol. 2017;19: 1497–1504.

[25] Villagarcia H, Morin A-C, Shpak ED, Khodakovskaya MV. Modification of tomato growth by expression of truncated ERECTA protein from Arabidopsis thaliana. J Exp Bot. 2012;63: 6493–6504. 10.1093/jxb/ers305 23096000

[26] Paz MM, Shou H, Guo Z, Zhang Z, Banerjee AK, Wang K. Assessment of conditions affecting Agrobacterium-mediated soybean transformation using the cotyledonary node explant. Euphytica. 2004;136: 167–179.

[27] Paz MMM, Wang K. Soybean transformation and regeneration using half-seed explant. US7473822B1, 2009. https://patents.google.com/patent/US7473822B1/en

[28] Paz MM, Martinez JC, Kalvig AB, Fonger TM, Wang K. Improved cotyledonary node method using an alternative explant derived from mature seed for efficient Agrobacterium-mediated soybean transformation. Plant Cell Rep. 2006;25: 206–213. 10.1007/s00299-005-0048-7 16249869

[29] SAS Functions by Example, Second Edition.: 62.

[30] Barrs HD, Weatherley PE. A Re-Examination of the Relative Turgidity Technique for Estimating Water Deficits in Leaves. Aust J Biol Sci. 1962;15: 413–428. 10.1071/bi9620413

[31] Chaudhary J, Patil GB, Sonah H, Deshmukh RK, Vuong TD, Valliyodan B, et al Expanding Omics Resources for Improvement of Soybean Seed Composition Traits. Front Plant Sci. 2015;6 10.3389/fpls.2015.01021 26635846 PMC4657443

[32] Kosentka PZ, Zhang L, Simon YA, Satpathy B, Maradiaga R, Mitoubsi O, et al Identification of critical functional residues of receptor-like kinase ERECTA. J Exp Bot. 2017;68: 1507–1518. 10.1093/jxb/erx022 28207053 PMC5441908

[33] Yokoyama R, Takahashi T, Kato A, Torii KU, Komeda Y. The Arabidopsis ERECTA gene is expressed in the shoot apical meristem and organ primordia. Plant J. 1998;15: 301–310. 10.1046/j.1365-313x.1998.00203.x 9750343

[34] Xing HT, Guo P, Xia XL, Yin WL. PdERECTA, a leucine-rich repeat receptor-like kinase of poplar, confers enhanced water use efficiency in Arabidopsis. Planta. 2011;234: 229–241. 10.1007/s00425-011-1389-9 21399949

[35] Qi Y, Sun Y, Xu L, Xu Y, Huang H. ERECTA is required for protection against heat-stress in the AS1/AS2 pathway to regulate adaxial–abaxial leaf polarity in Arabidopsis. Planta. 2004;219: 270–276. 10.1007/s00425-004-1248-z 15034716

[36] Jordá L, Sopeña-Torres S, Escudero V, Nuñez-Corcuera B, Delgado-Cerezo M, Torii KU, et al ERECTA and BAK1 Receptor Like Kinases Interact to Regulate Immune Responses in Arabidopsis. Front Plant Sci. 2016;7 10.3389/fpls.2016.00897 27446127 PMC4923796

[37] Patel D, Basu M, Hayes S, Majláth I, Hetherington FM, Tschaplinski TJ, et al Temperature-dependent shade avoidance involves the receptor-like kinase ERECTA. Plant J. 2013;73: 980–992. 10.1111/tpj.12088 23199031

[38] Kasulin L, Agrofoglio Y, Botto JF. The receptor-like kinase ERECTA contributes to the shade-avoidance syndrome in a background-dependent manner. Ann Bot. 2013;111: 811–819. 10.1093/aob/mct038 23444123 PMC3631326

[39] Ren S, Lyle C, Jiang G, Penumala A. Soybean Salt Tolerance 1 (GmST1) Reduces ROS Production, Enhances ABA Sensitivity, and Abiotic Stress Tolerance in Arabidopsis thaliana. Front Plant Sci. 2016;7 10.3389/fpls.2016.00445 27148284 PMC4826999

[40] Halford N. Plant Biotechnology: Current and Future Applications of Genetically Modified Crops. John Wiley & Sons; 2006.

[41] Use of GmRD22-like genes to protect against abiotic stress. [cited 2 Apr 2020]. http://www.freepatentsonline.com/20080184385.pdf

[42] Wang H, Zhou L, Fu Y, Cheung M-Y, Wong F-L, Phang T-H, et al Expression of an apoplast-localized BURP-domain protein from soybean (GmRD22) enhances tolerance towards abiotic stress. Plant Cell Environ. 2012;35: 1932–1947. 10.1111/j.1365-3040.2012.02526.x 22548236

[43] Kapilan R, Vaziri M, Zwiazek JJ. Regulation of aquaporins in plants under stress. Biol Res. 2018;51 10.1186/s40659-018-0152-0 29338771 PMC5769316

[44] Soybean water channel protein gene GmPIP1; The application of 2. CN103266130B, 2015. https://patents.google.com/patent/CN103266130B/en

