# Supplementary figures and images for: Modification of soybean growth and abiotic stress tolerance by expression of truncated ERECTA protein from Arabidopsis thaliana
Source: PLoS One. 2020 May 19;15(5):e0233383. doi: 10.1371/journal.pone.0233383 (PMC7236981; doi:10.1371/journal.pone.0233383)

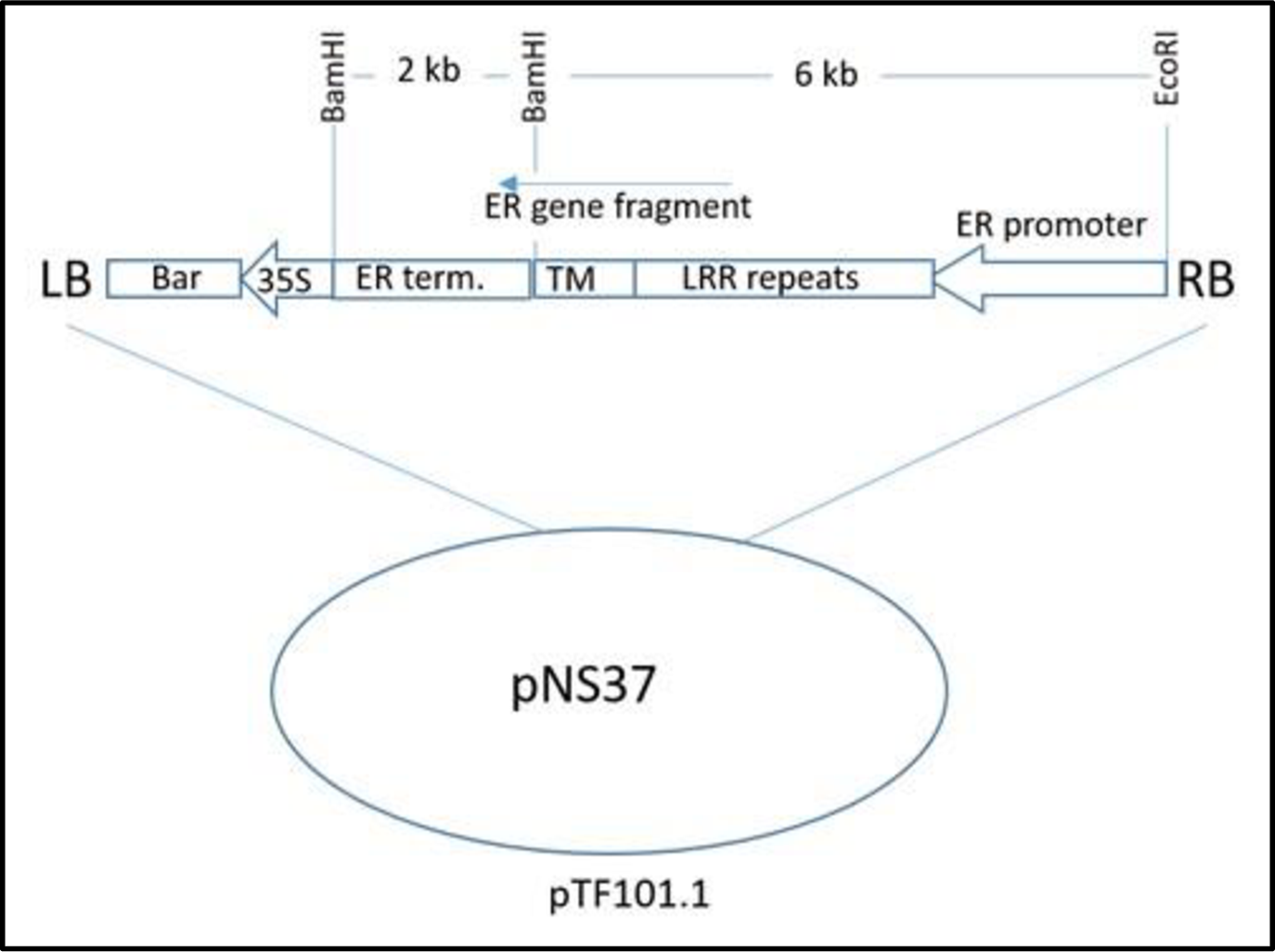

Supplement: S1 Fig — The pNS37 construct in pTF101.1 backbone is shown. The 6 kb EcoR1 –BamH1 fragment from pESH454 was cloned between EcoR1 and BamH1 sites of pTF101.1 followed by introduction of 2 kb BamH1 fragment to build pNS37. The ER gene fragment consists of exons and introns for LRR repeats and transmembrane (TM) region. There is a stop codon immediately after TM sequence followed by BamH1 site and ER terminator. The pTF101.1 vector contains 2 x 35S promoter driven Bar gene as the selection marker. (TIF) [file pone.0233383.s001.tif]

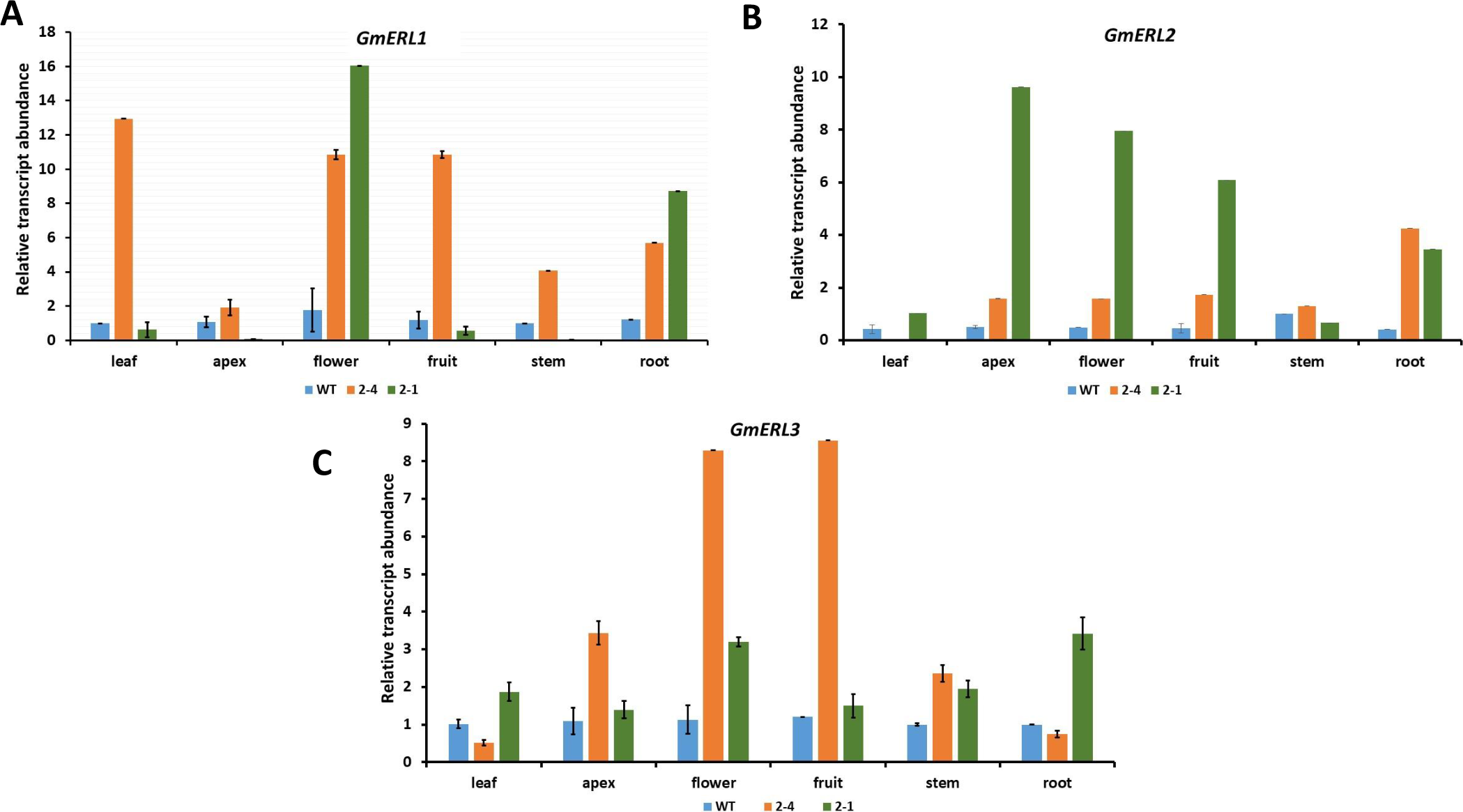

Supplement: S2 Fig — 18S was used as an internal control. Samples of leaves, apices, stem and root were collected from 21-day-old plants, flowers were collected from 35-day-old plants and siliques derived from 45-day-old plants. Results are shown as means ±SE of three biological replicates. (TIF) [file pone.0233383.s002.tif]

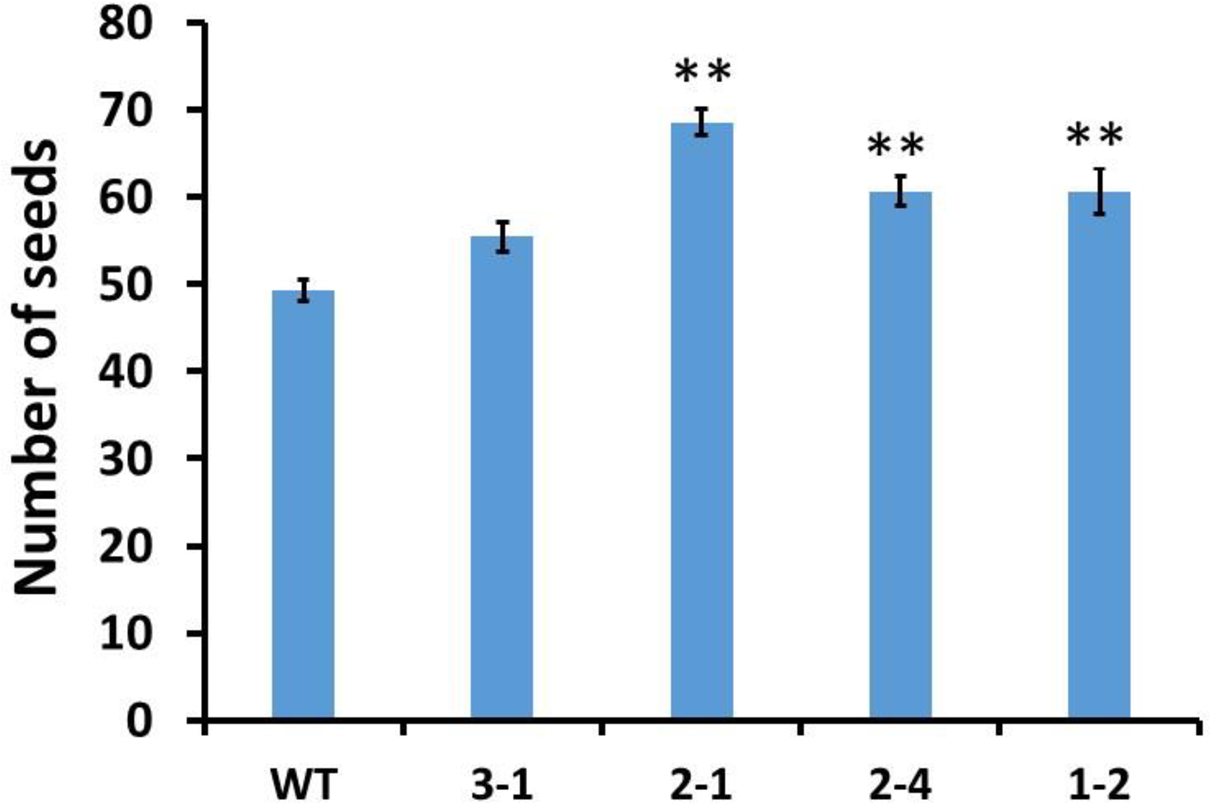

Supplement: S3 Fig — n = 10; **P <0.01; *P <0.05. Results are shown as means ±SE. (TIF) [file pone.0233383.s003.tif]

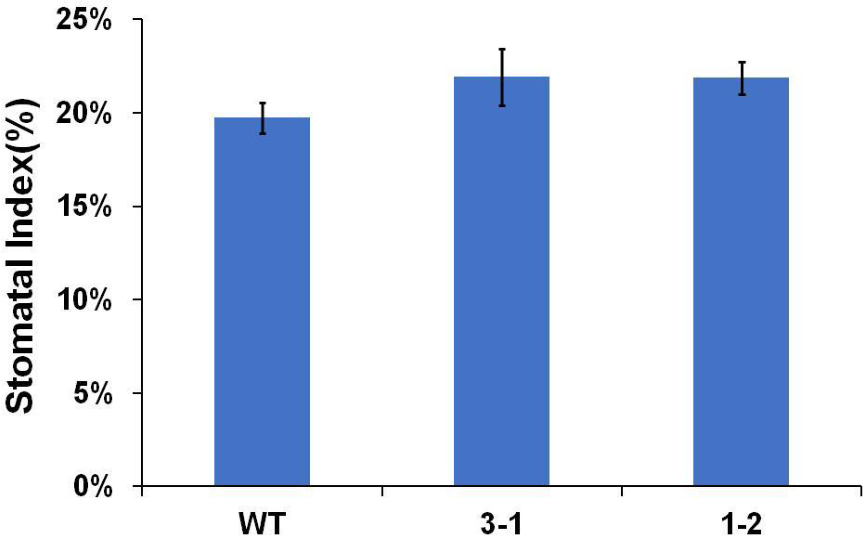

Supplement: S4 Fig — Six leaves were sampled and analyzed for each line. Values are mean ± SE. (TIF) [file pone.0233383.s004.tif]

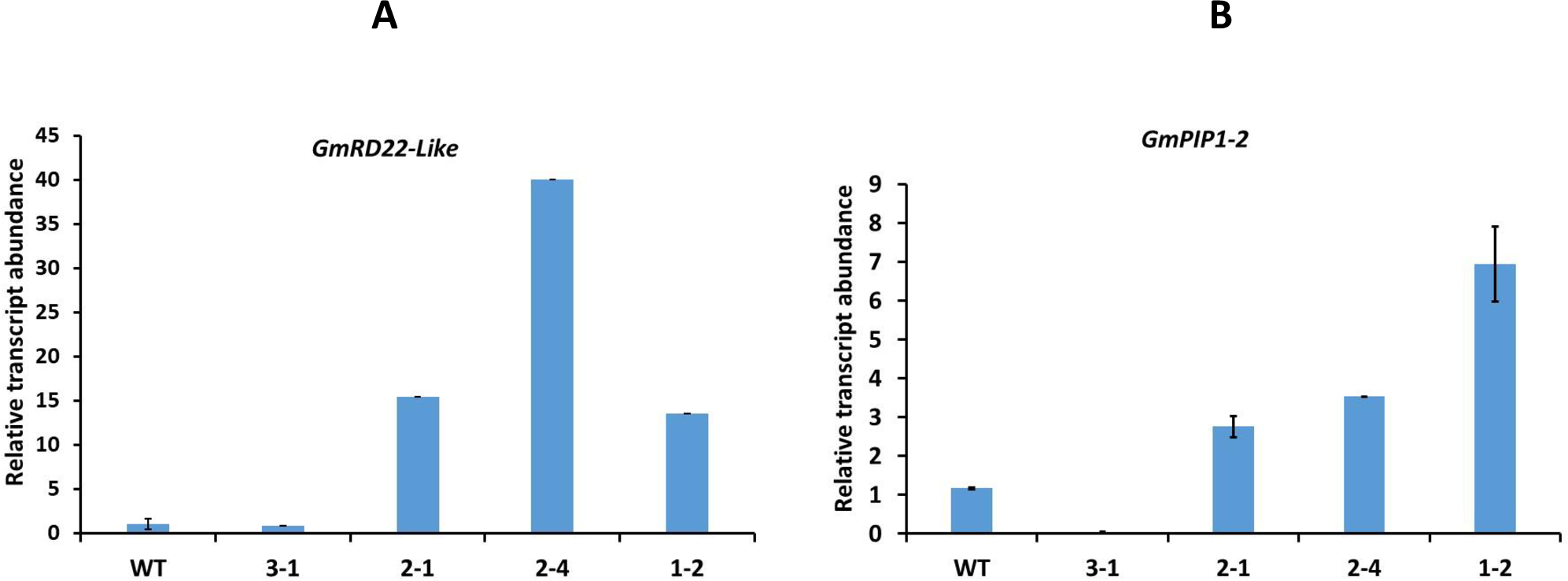

Supplement: S5 Fig — Leaves were collected from 28-day-old wild type and of transgenic plants grown in conditions of water deficit for 7 days. 18S was used as an internal control. Results are shown as means ±SE of three biological replicates. (TIF) [file pone.0233383.s005.tif]

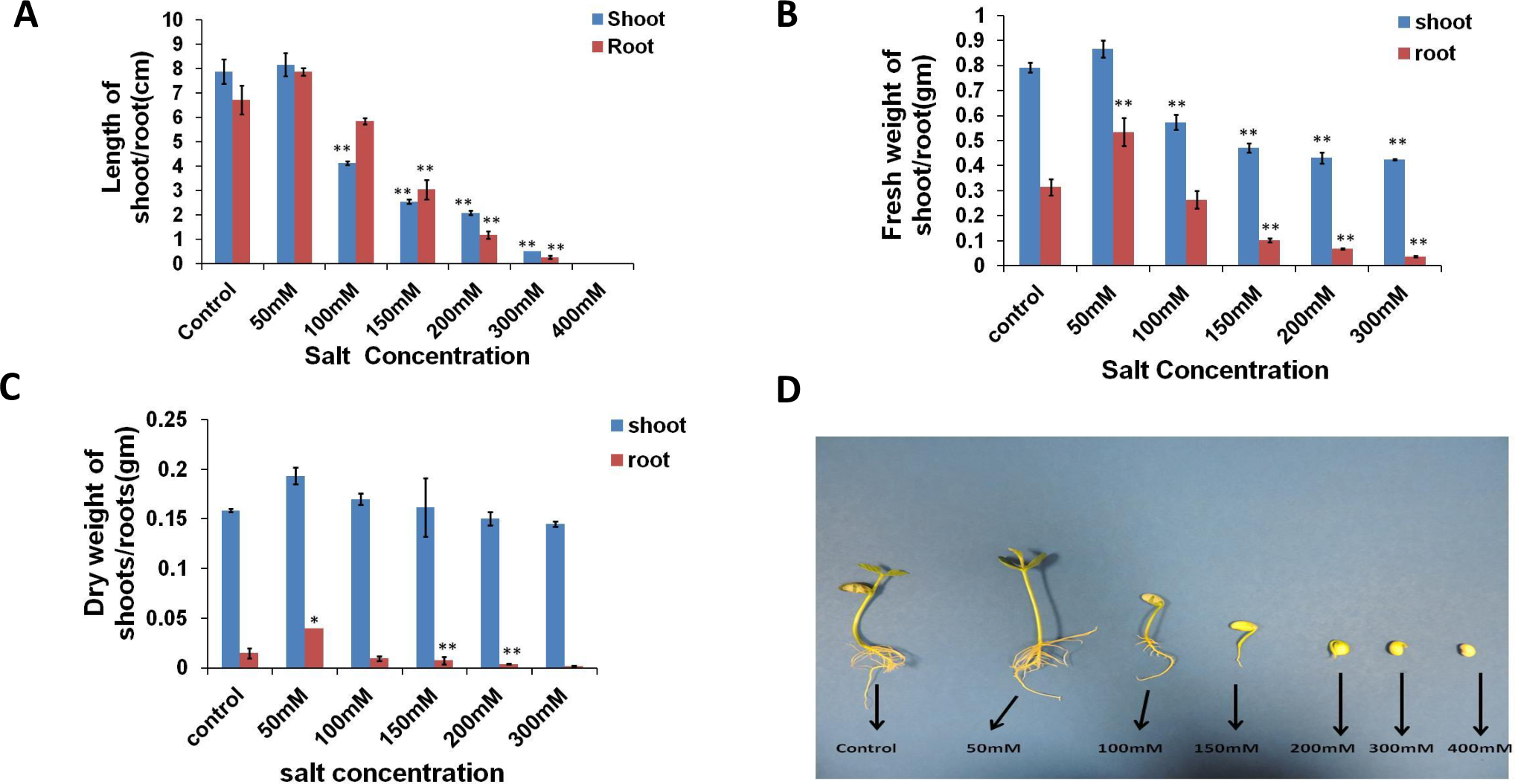

Supplement: S6 Fig — Phenotypical comparison of 10-day-old soybean wild-type seedlings unexposed to salt stress and seedlings exposed to different concentrations of NaCl (50mM, 100mM, 150mM, 200mM, 300mM and 400mM) (A, B, C) n = 10; **P <0.01; *P <0.05. Results are shown as means ±SE. (D) Photograph of 10-day-old wild type seedlings grown without NaCl supplement (control) and grown in medium supplemented with a wide range of NaCl concentrations. (TIF) [file pone.0233383.s006.tif]

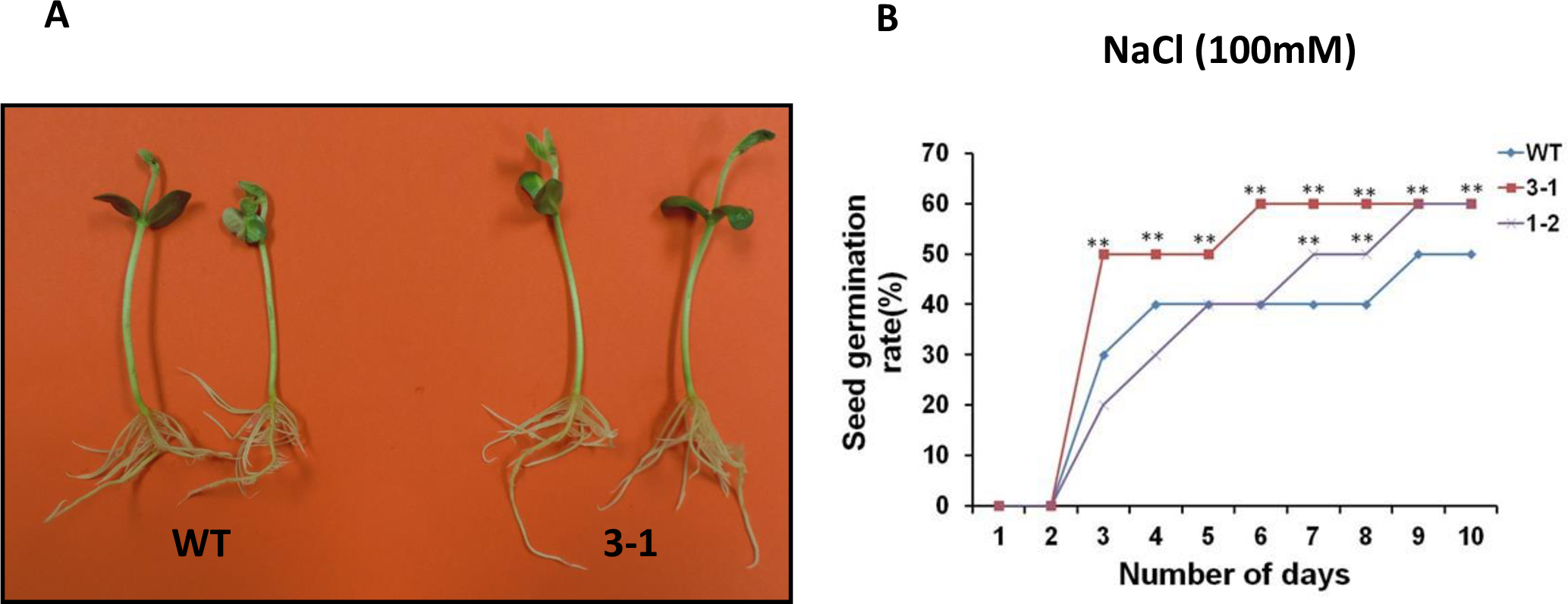

Supplement: S7 Fig — (A) The phenotype of wild type and transgenic AtERpro: AtΔKinase seedlings (line 3–1) exposed to salt stress conditions (100mM NaCl). (B) AtERpro:AtΔKinase transgenic plants exhibited higher germination rate on medium supplemented with 100mM of NaCl compared to wild-type. (TIF) [file pone.0233383.s007.tif]
